# Supplementary material for: Structures of ABCG2 under turnover conditions reveal a key step in the drug transport mechanism
Source: Nat Commun. 2021 Jul 19;12:4376. doi: 10.1038/s41467-021-24651-2 (PMC8289821; doi:10.1038/s41467-021-24651-2)
Supplement: Supplementary file 1 — Supplementary Information [file 41467_2021_24651_MOESM1_ESM.pdf]

Supplementary information

Structures of ABCG2 under turnover conditions reveal a key step in the drug transport mechanism

Qin Yu[1], Dongchun Ni[2], Julia Kowal[1], Ioannis Manolaridis[1], Scott M. Jackson[1], Henning Stahlberg[2], Kaspar P. Locher[1]*

[1]Institute of Molecular Biology and Biophysics, Department of Biology, ETH Zürich, Zürich, Switzerland.
[2]Center for Cellular Imaging and NanoAnalytics (C-CINA), Biozentrum, University of Basel, Basel, Switzerland. Current address: Laboratory of Biological Electron Microscopy, Institute of Physics, SB, EPFL, Lausanne, Switzerland.

*E-mail for correspondence: locher@mol.biol.ethz.ch

Supplementary Figures

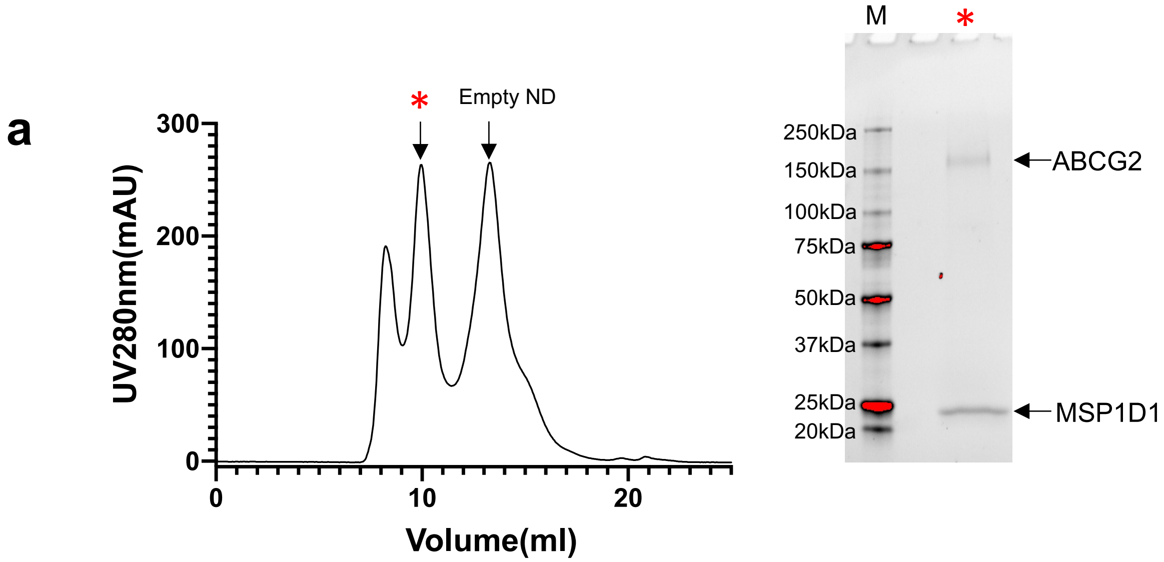

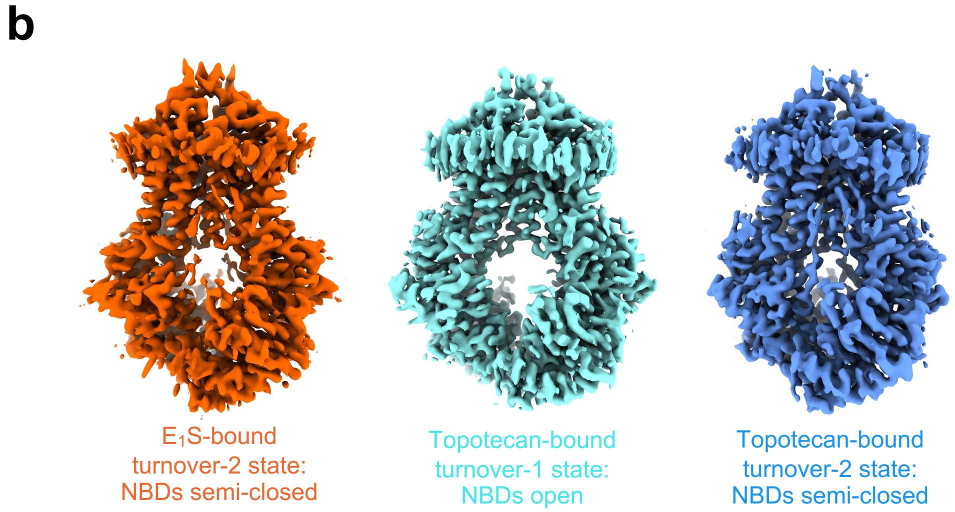

Supplementary Figure 1. Sample preparation and cryo-EM maps
a Left, the preparative gel filtration profile of nanodisc-reconstituted ABCG2. Empty ND indicates the peak for empty nanodisc. Right, representative SDS–PAGE analysis of main peak obtained in gel filtration. No reducing agent was used and ABCG2 runs as a disulfide-linked dimer. M shows marker proteins, with masses indicated on the left. The red asterisk shows the peak fraction analyzed in the gel and used for EM grid preparation. Source data are provided in the related Source Data file. Experiment was repeated 3 times with similar results. **b** Non-symmetrized cryo-EM maps of turnover states.

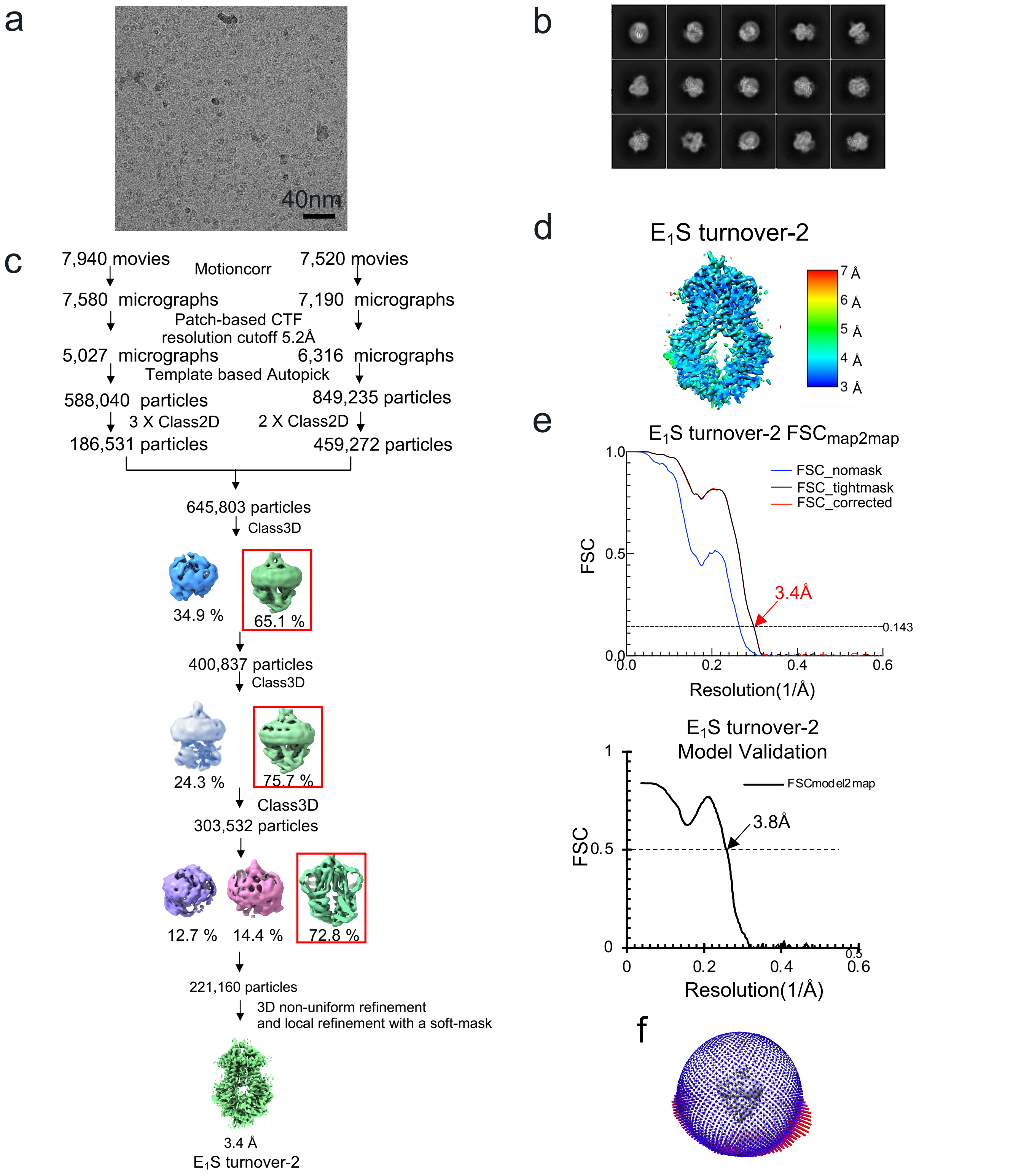

Supplementary Figure 2. Cryo-EM data processing and model validation of E_1S turnover structure.

a A representative motion-corrected 2D micrograph of E_1S turnover sample among 15,460 images collected. Black scale bar, 400 Å. **b** Representative 2D classes. **c** Flowchart of data processing of E_1S turnover sample. Red boxes indicate classes of particles selected for the next round. **d** Local resolution of E_1S turnover-2. **e** Map and model validation of E_1S turnover-2. Source data are provided in the related Source Data file. FSC is short for Fourier shell correlation. **f** Angular distribution plot for the final reconstruction of E_1S turnover-2 from RELION.

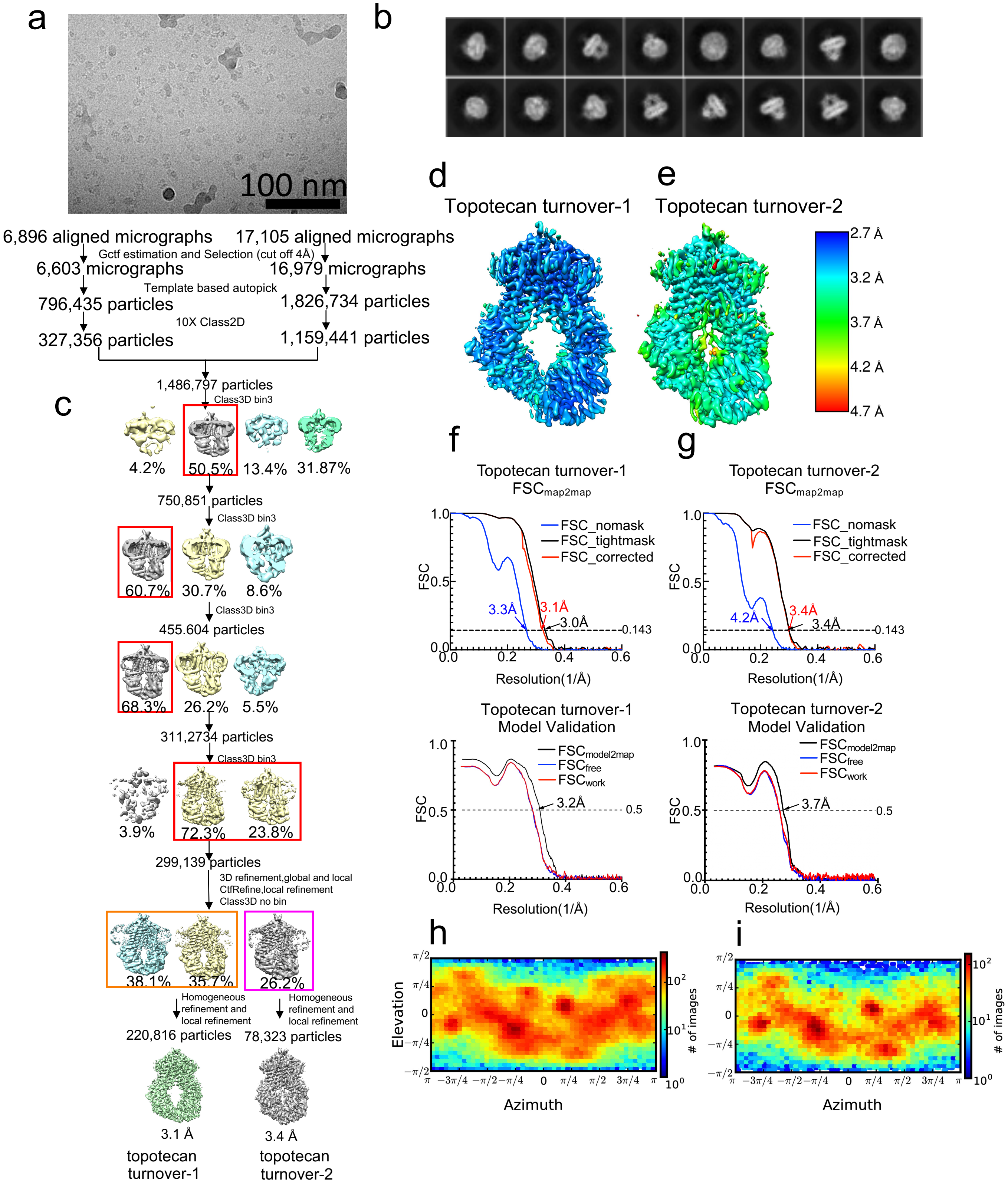

Supplementary Figure 3. Cryo-EM data processing and model validation of topotecan turnover structures.

a A representative motion-corrected 2D image of topotecan turnover sample among 24,001 images collected. **b** Representative 2D classes. **c** Flowchart of data processing of topotecan turnover sample. Red boxes indicate classes of particles selected for the next round. **d** and **e** local resolution plot for topotecan turnover structures. **f** and **g** map and model validation of topotecan turnover structures. Source data are provided in the related Source Data file. **h** and **i** Angular distribution plot of topotecan turnover structures from CryoSPARC.

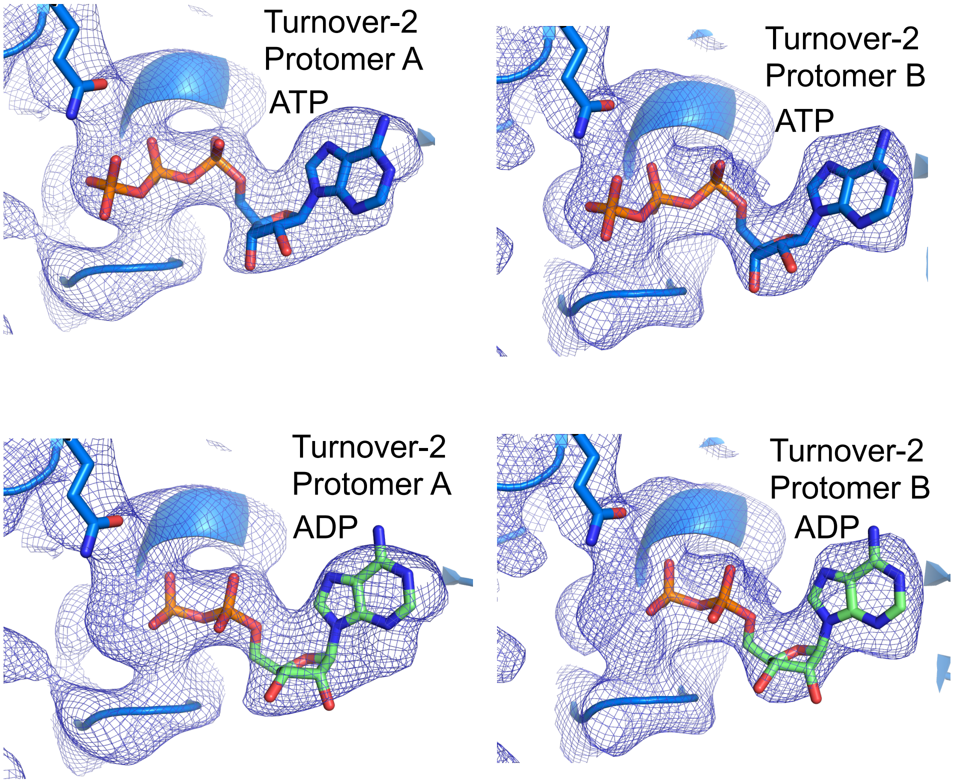

Supplementary Figure 4. EM maps of nucleotide-binding sites in turnover-2 state
The structure of ABCG2 in topotecan-bound turnover-2 state is shown as ribbons and sticks. EM density is shown as a blue mesh. ATP (top, shown in blue) or ADP (bottom, shown in green) were built into the density at the nucleotide-binding site of each ABCG2 protomer. While ATP fits the density, ADP leaves unexplained density.

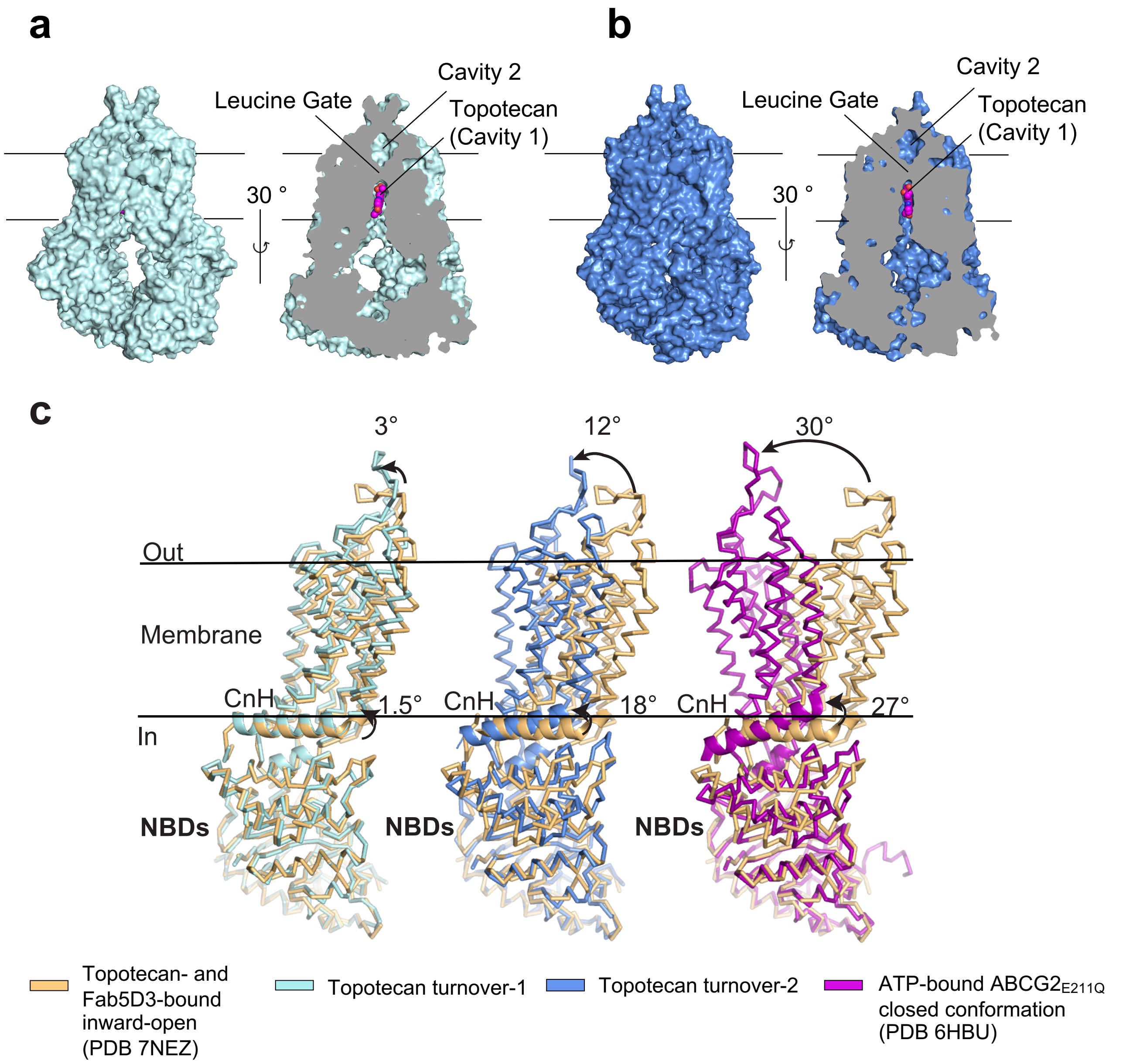

Supplementary Figure 5. Conformational changes in ABCG2

a Surface representation (left) and vertical slice (right) of topotecan turnover-1 structure. Bound topotecan in cavity is shown as spheres and labeled. The leucine gate and cavity 2 are also labeled. **b** Similar to **a** but showing the topotecan turnover-2 structure. **c** Structural changes within ABCG2 protomers upon superposition of the NBDs (nucleotide-binding domains). Bound 5D3-Fab is omitted for clarity. The structures are color-coded. The distinct rigid body rotations of the TMDs (transmembrane domains) relative to the TMD of the topotecan- and 5D3Fab-bound structure are shown by arrows. Similarly, the rotations of the connecting helices (CnH) are indicated.

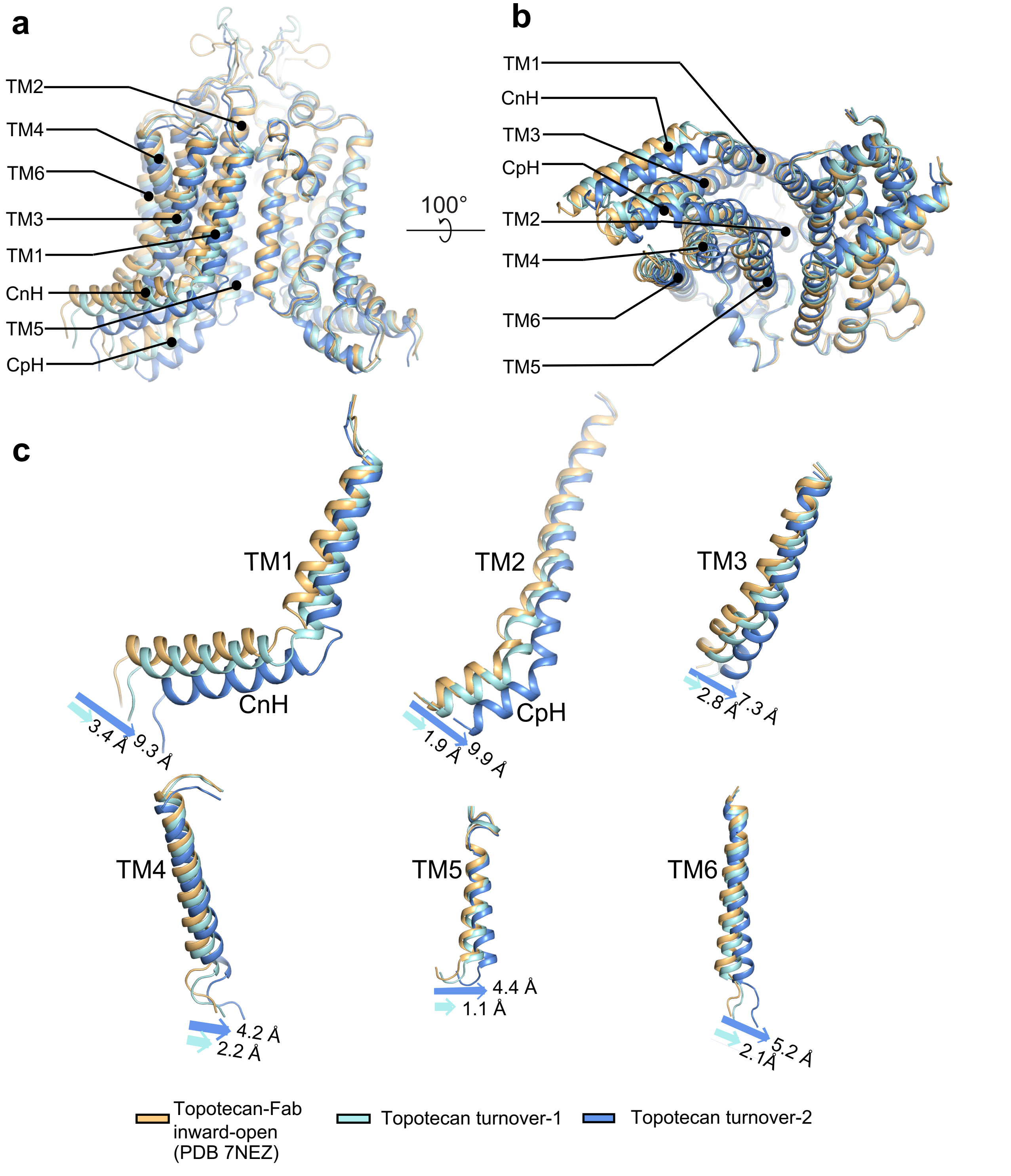

Supplementary Figure 6. Conformational changes within TMDs

ABCG2 structures (color coding in panel **c**) are shown upon superposition of one of the TMDs (right side in panels **a** and **b**) to visualize the magnitude of the structural changes in the TMD dimer. **a** Side view of the TMDs after superposition. Transmembrane (TM) helices, coupling helices (CpH) and connecting helices (CnH) are numbered and labeled. NBDs are omitted for clarity. **b** Similar to **a** but viewed from the inside of the cell. **c** Close-up view of each TM helix in the non-superimposed TMD. Shifts at the cytoplasmic end of each TM helix or CpH or CnH are indicated.

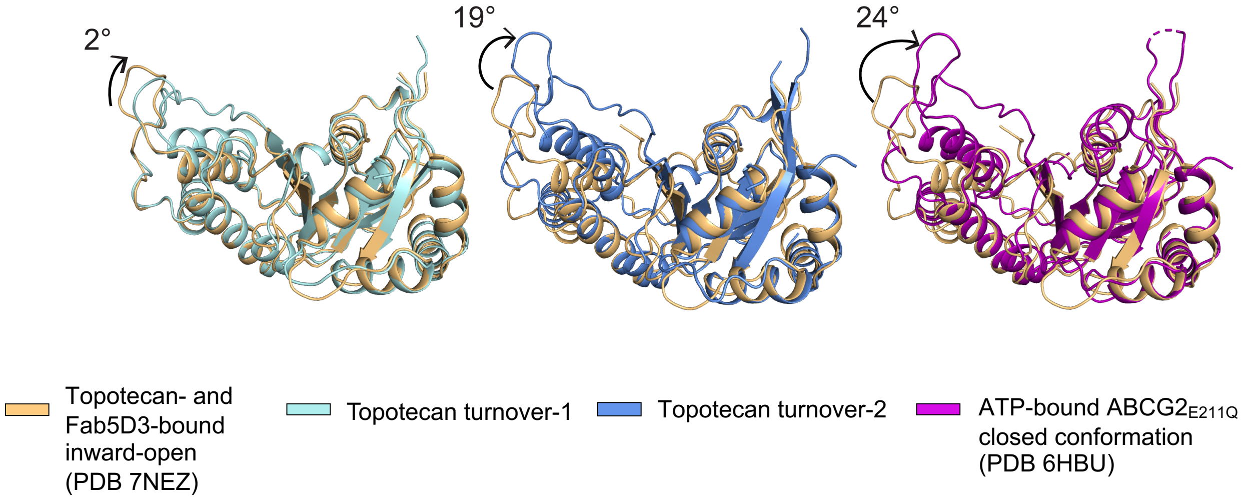

Supplementary Figure 7 Subdomain rotations within NBDs

Superpositions of RecA-like subdomain in NBDs of ABCG2, illustrating the rotation of the helical subdomain relative to the helical subdomain of topotecan- and Fab-bound inward-open ABCG2. The structures are color-coded and the degree of the rotation is indicated.

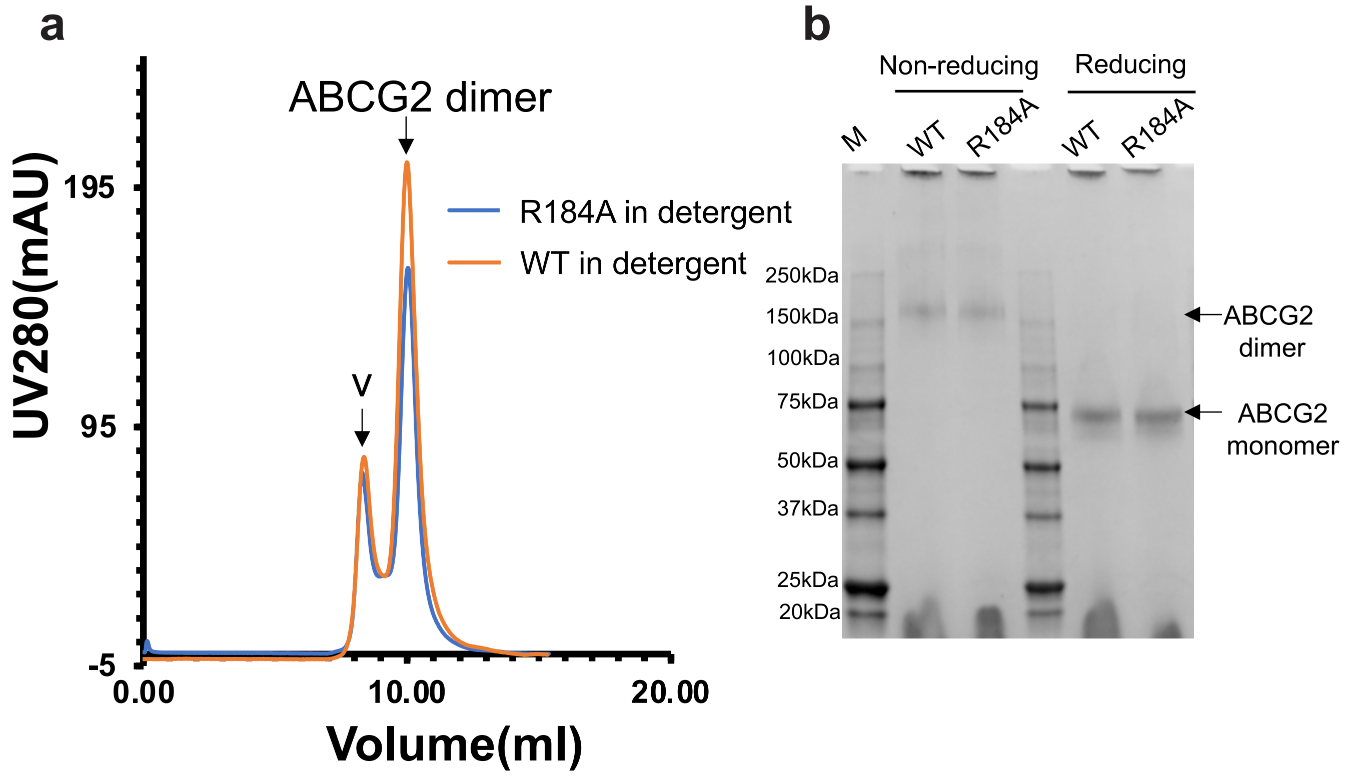

Supplementary Figure 8 Biochemical characterization of wild type ABCG2 and ABCG2$_{R184A}$

a Preparative gel filtration profile of WT ABCG2 and ABCG2$_{R184A}$ in detergent solution. Void peak (indicated by “v”) and ABCG2 dimer peak are indicated by arrows. **b** A Representative SDS–PAGE analysis of ABCG2 reconstituted in proteoliposomes. Both reducing and none-reducing conditions were used. WT and R184A run as covalently (disulfide) linked dimers in non-reducing conditions and as monomers in reducing conditions. M shows marker proteins, with masses indicated on the left. Experiment was repeated 3 times with similar results. Source data for panel a and b are provided in the related Source Data file.

a

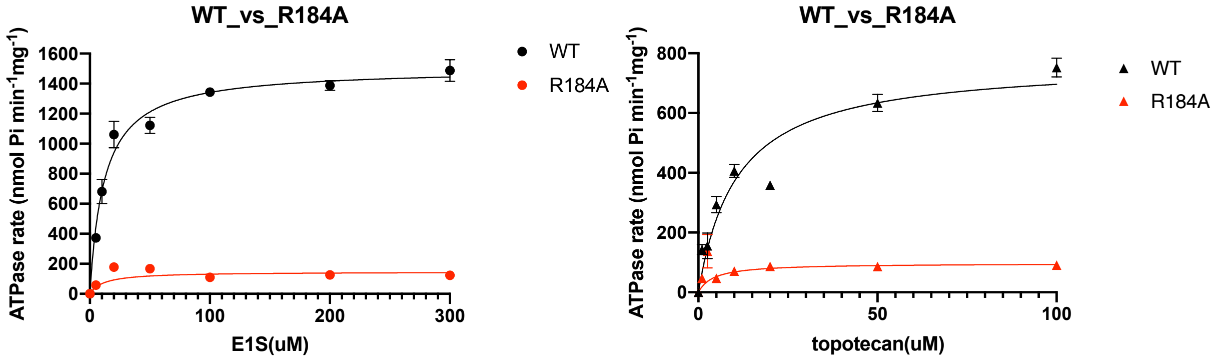

b

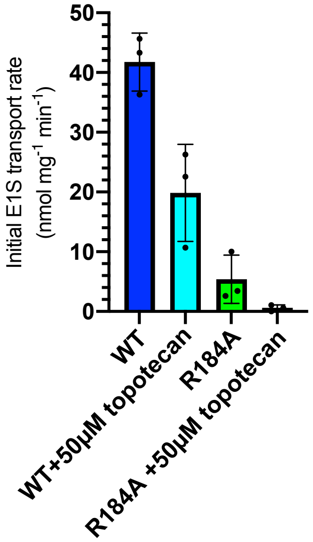

**Sample**	**Initial rate (mol E_1S/mol G2 dimer/s)**
ABCG2 WT	0.10 ± 0.01
ABCG2 WT+50µM topotecan	0.048 ± 0.02
ABCG2 R184A	0.013 ± 0.01
ABCG2 R184A +50µM topotecan	-

Supplementary Figure 9 ATPase and transport activity of wild type ABCG2 and ABCG2$_{R184A}$ in proteoliposomes

a Stimulation of ATPase rate of WT ABCG2 and ABCG2$_{R184A}$ by E_1S (left) and topotecan (right). The basal ATPase activity was normalized to zero. Error bars show s.d., each point represents the mean rate and the experiment was performed 3 times independently with the same batch of liposomes.

b Left, initial E_1S transport rates of wild type ABCG2 and ABCG2$_{R184A}$ variant. Error bars show s.d.. Each bar represents the mean rate. The experiment was performed 3 times independently with the same batch of liposomes. Right, table of initial ATPase rates of experiments shown in the left panel. Source data for panel a and b are provided in the related Source Data file.

Supplementary Tables

Structure	topotecan- and 5D3-Fab-bound inward-open conformation (PDB: 7NEZ)	topotecan turnover-1	E_1S turnover-2	Closed conformation (PDB: 6HBU)
RMSD relative to topotecan turnover-2 conformation	2.7 Å	2.1 Å	0.5 Å	3.3 Å

Supplementary table 1. Overall R.M.S.D between ABCG2 structures of different conformations

Substrate	EC_{50} (μM) (ATPase)
Mitoxantrone	18.3 ± 2.2[a]
Topotecan	11.7 ± 0.3[a]
Tariquidar	0.08 ± 0.01[a]
E_1S	15.7±0.9 [b]
E_1S+ Fab	18.0±4.7[b]

Supplementary table 2. EC_{50} of ATPase stimulation of ABCG2 by substrates. The data is combined from previous study to allow direct comparison.
[a]Data taken from[23]
[b]Data taken from[19]

Cryo-EM data collection, refinement and validation statistics			
Data collection and processing	E_1S turnover	Topotecan turnover	
Map name	turnover-2	turnover-1	turnover-2
Energy filters & detector	Quantum-LS / K2	BioQuantum / K3	
Energy window (eV)	20	20	
Magnification (nominal)	165,000 x	130,000 x	
Voltage (kV)	300	300	
Electron exposure per movie (e^-/Å^2)	50	58	
Electron exposure per frame (e^-/Å^2/frame)	1.25	1.45	
Defocus range (µm)	0.8-2.8	0.6-2.0	
Pixel size (Å)	0.82	0.66	
symmetry	C1	C1	
Initial particle images (No.)	645,803	2,623,169	
Final particle images (No.)	221,160	220,816	78,323
Map resolution (Å)	3.40	3.13	3.34
FSC threshold	0.143	0.143	0.143
Map resolution range (Å)	2.7-20.0	2.65-20	3-20
Model refinement			
Initial model used	6HCO	6HCO	6HCO
Model resolution(Å)	3.8	3.2	3.5
FSC threshold	0.5	0.5	0.5
model resolution range (Å)	3.8-30	3.2-254	3.5-254
Map sharpening B factor (Å²)	-118	-106.5	92.8
Model composition			
Non-hydrogen atoms	9,148	8,999	9,195
protein residues	1,150	1,138	1,144
Ligands	ATP: 2 CLR:4 E_1S:1	ATP: 2 CLR:2 Topotecan:1	ATP: 2 CLR: 4 Topotecan:1 PLC: 2
B factors (Å²)			
protein	65.86/155.94/96.41	17.21/65.49/35.70	45.05/100.99/64.77
ligand	45.56/118.32/83.62	18.42/48.88/31.46	20.00/77.34/58.80
R.m.s.d deviations			
Bond lengths (Å)	0.008	0.006	0.009
bond angles (°)	0.875	0.655	0.721
Validation			
MolProbity score	1.75	1.17	1.53
Clash score	6.54	2.32	3.98
Poor rotamers (%)	0.41	0.52	0.41
Ramachandran plot			
Favored (%)	94.18	97.15	95.04
Allowed (%)	5.29	2.85	4.79
Disallowed (%)	0.53	0	0.18
PDB	7OJ8	7OJH	7OJI
EMDB	EMD-12939	EMD-12951	EMD-12952

Supplementary table 3. Cryo-EM data collection, refinement and validation statistics
